# “It’s Cool, Modifying and All, but I Don’t Want Anything Blowing Up on Me:” A Focus Group Study of Motivations to Modify Electronic Nicotine Delivery Systems (ENDS)

**DOI:** 10.3390/ijerph182211735

**Published:** 2021-11-09

**Authors:** Zachary B. Massey, Robert T. Fairman, Victoria Churchill, David L. Ashley, Lucy Popova

**Affiliations:** 1 School of Journalism, University of Missouri, Columbia, MO 65211-1200, USA; zbmassey@missouri.edu; 2 School of Public Health, Georgia State University, Atlanta, GA 30302-3995, USA; rfairman1@student.gsu.edu (R.T.F.); vchurchill1@student.gsu.edu (V.C.); dashley4@gsu.edu (D.L.A.)

**Keywords:** ENDS modifications, vaping, e-liquids, cannabis, coils

## Abstract

Introduction: Modifications to electronic nicoti ne delivery systems (ENDS) can pose health risks to users. This study explored users’ motivations for modifying ENDS devices and how perceived risks of modifications influenced modification behaviors as product availability and device characteristics changed over time. Method: We conducted nine focus groups (February–June 2020) with 32 current ENDS users (18+, used ENDS in the past 30 days, and had been using ENDS for more than 2 months). Results: Participants primarily modified ENDS devices to improve their experiences, such as experimenting with flavor, controlling nicotine levels, or using cannabis products with ENDS. Another reason for modifying was routine maintenance to ensure a satisfactory experience, including maintaining coils and keeping batteries charged. The broader availability of ENDS products shifted modification behaviors over time, with newer devices making some modifications (e.g., coil replacement) easier and making more intricate modifications (e.g., building coil from scratch) less common. Participants were aware of modification dangers and cited perceived risk as the reason for avoiding certain modifications, such as battery alterations. Conclusions: Modifications of ENDS are ongoing and evolving among users and should be considered by the Food and Drug Administration (FDA) and other regulatory decision-makers as product authorization reviews are conducted and product standards are developed.

## 1. Introduction

Since 2010, the use of electronic nicotine delivery systems (ENDS) in the United States has increased dramatically [1,2,3]. ENDS are devices where a battery-heated coil aerosolizes e-liquids comprising propylene glycol (PG) and/or vegetable glycerin (VG) and other chemicals, such as nicotine and flavorings [4,5,6,7]. ENDS include a wide variety of devices commonly called vapes, e-cigarettes, e-hookahs, drippers, tanks, pod mods, and disposables [8,9]. These devices are now widely available in the United States [8,9,10].

As ENDS have evolved from the original “cigalike” products, they have become more adjustable, allowing users greater control over product characteristics [5,11]. Some users also modify ENDS in ways unintended by manufacturers [12,13]. For this study, we use a broad definition of *modification* as both product tampering unintended by the manufacturers, as well as the customization, adjustment, and user choice of e-liquid or accessories made within manufacturer specifications [12,13]. Both types of modification are important to consider in determining whether a product should be authorized for marketing because products should be evaluated as actually used by consumers, and this may include a wide range of characteristics. ENDS modifiability appeals to users [12,13,14]. However, some ENDS modifications pose health risks, including battery explosions [15,16], higher levels of toxic emissions from increasing power to the coil [17,18], and severe lung injury from inhaling harmful additives [19,20]. Given the popularity of ENDS and the health risks associated with modifications, more research is needed to understand what motivates users to modify ENDS and how perceived risks of modification influence use behavior.

To date, little is known about what motivates consumers to modify ENDS devices. The Population Assessment of Tobacco and Health (PATH) survey asks whether participants had ever modified an e-cigarette [21]. To the best of our knowledge, PATH modification data have not been published. Cross-sectional research has detailed the prevalence of specific modification behaviors, such as dripping e-liquid on coils [22,23,24] or adapting ENDS for drug use [7,25,26,27]. However, these studies have not explored how users perceive the risks of modifying ENDS as product availability and design characteristics evolve.

Our research program aimed to understand users’ modification behaviors. Previously, we interviewed “ENDS enthusiasts” and found that they modified device coils, batteries, and e-liquids in order to produce larger clouds, increase nicotine delivery, and achieve a desired flavor [12]. These interviews revealed that changes in product design over time had decreased the prevalence of ENDS modifications [12]. However, these enthusiasts were highly interested in ENDS and had extensive experience modifying devices. Our content analysis of YouTube videos documented user modifications in a diverse range of ENDS products, suggesting ongoing popularity for ENDS modifications [13]. However, there is little descriptive research documenting ENDS modification behaviors of regular or non-enthusiast users. In this study, the inclusion criteria were defined as a current ENDS user (i.e., used in the last 30 days) who had been using ENDS for at least 2 months and was willing to discuss modifying devices. Questions remain regarding what modifications regular ENDS users perform and why and how modification behaviors may have changed as regulatory and commercial environments for ENDS products have evolved. Understanding the modification behaviors of regular users is timely because the Food and Drug Administration (FDA) is currently reviewing more than six million pre-market tobacco applications from ENDS manufacturers.

To provide insight on this topic, we conducted focus groups with current ENDS users to understand what motivated them to modify ENDS devices and how perceived risks of modifications influenced behaviors. We explored how ENDS modifications have evolved due to product availability and device characteristic changes and how users learned about modifications.

## 2. Methods

We conducted focus groups using a qualitative description approach. Qualitative description seeks a “rich, straight description” [28] using structured interviews to collect qualitative data [28]. Analysis remains close to the data, with the results reported in the participants’ own language [28,29]. Qualitative description is appropriate for focus groups [28] where participants speak freely and provide different perspectives on various topics [30]. This paper is organized according to the Standards of Reporting Qualitative Research [31].

### 2.1. Research Team and Reflexivity

The research company John Snow Inc. (JSI), Atlanta, GA, USA, recruited participants and ran focus groups. The JSI study lead was a Licensed Master Social Worker with a Master’s Degree in Public Health. Another JSI employee assisted. Together, the two JSI employees recruited and moderated all focus groups. Outside JSI, no members of the research team interacted with participants.

### 2.2. Participants and Procedures

Purposeful sampling [32] was used to identify and select current ENDS users. Inclusion criteria were current ENDS users (i.e., 18+, used ENDS in the past 30 days) who had been using for more than 2 months and were willing to talk about ENDS use behavior and about modifying ENDS. Participants were recruited in the United States using online (e.g., Facebook) and offline (e.g., flyers) strategies. In contrast to our previous study of ENDS enthusiasts, many of whom worked in e-cigarette stores [12], our sampling criteria were intentionally broad to recruit regular ENDS users. Interested individuals completed online screening about demographics and tobacco use, and those eligible were contacted to participate in focus groups.

Nine focus groups were conducted with 32 participants in February–June 2020. Three in-person groups were held in Atlanta, GA, USA. After March 2020, the remaining six groups were conducted using videoconferencing software due to COVID-19 with participants from across the country. We do not expect Georgia participants to differ in any substantial way from participants in other U.S. states. The JSI study lead moderated focus groups with a structured interview guide using open-ended questions [28]. The moderator guide was developed based on interviews with ENDS users [12], previous literature, and expert consensus by our interdisciplinary team of collaborators with substantial expertise in ENDS use behavior. Focus groups lasted between 38 and 81 min (median 71 min). The number of participants ranged between one (in the last in-person focus group due to no-shows right at the beginning of COVID-19) and six (median 3). Focus groups were audio-recorded. Each participant received USD 50 compensation. The Georgia State University Institutional Review Board approved the study, and JSI obtained electronic (screeners) and verbal (interviews) consent from all participants.

### 2.3. Data Analysis

Focus group discussions were transcribed and anonymized by JSI before dissemination to the research team. Transcripts were analyzed using a qualitative description approach [28,29]. Specifically, Z.B.M. read the transcripts and developed an initial codebook based on themes that closely followed participants’ answers about ENDS use behavior. Next, the research team met to discuss coding themes and refine codes. R.T.F. and V.C. independently coded two focus group transcripts. All coding discrepancies were discussed and resolved, and the codebook was updated and revised. R.T.F. and V.C. then split and coded the remaining transcripts in NVivo 13 [33]. Z.B.M., R.T.F., V.C., L.P., and D.L.A. reviewed coded transcripts, wrote summaries of the results of each code, and then met to discuss those results. Z.B.M. then synthesized the summaries.

## 3. Results

Table 1 shows all demographic and tobacco use characteristics. The sample was almost evenly split by sex (17 men, 15 women); 21 participants were White, 8 were Black. Of the total sample, 18 participants were current uses of e-cigarettes and smoked cigarettes, 7 were former smokers, and 7 were never smokers. Most (23) were using e-cigarettes daily.

Participants discussed various modifications to ENDS devices and e-liquids. We will first describe their reasons for making modifications (for improving user experience and maintaining the device), followed by reasons not to tamper with the products. We then finish by discussing sources of information for ENDS modifications.

### 3.1. Motivations to Modify: Improving User Experience

The most common reasons participants modified ENDS were to improve user experience by mixing e-liquid flavors to achieve the desired taste, altering nicotine levels to manage physiological needs, adding cannabis products (e.g., THC, tetrahydrocannabinol, and CBD, cannabidiol extracts) for perceived health benefits, and adjusting the wattage/voltage to control vapor clouds and throat hit.

#### 3.1.1. Modifying for Flavor and Nicotine: “As Long as You Can Get Your Nicotine Levels and Your Flavors, That’s the Most Important Thing”

Participants modified e-liquids to achieve the desired taste. Discussions revealed a preference for “fruity” e-liquid flavors such as “mango”, “watermelon”, and “apple”. One participant described mixing commercially sold candy-flavored e-liquids: “I’ve mixed Skittles and the Jolly brands of flavor together, and it came out pretty good” (male, 36). This participant also mixed e-liquids with perishable food items: “You can put some apple juice, orange juice, you want to mix it up. I did that before, I put a little bit of apple juice with the [e-]juices because the [e-]juice just tasted too strong. […] So you can just stick anything you want in there, if it could be liquidy you could stick it in there”.

Participants described the importance of varying nicotine levels to achieve a satisfactory hit. One participant described using different levels at different times. “Sometimes you need that quick hit and then sometimes you want to just like pace it out” (male, 29). Another participant (male, 23) described how friends modified ENDS with a “bigger piece of cotton so they can get a bigger cloud out of it or just vape more of the juice at one hit”. He then explained how changing inhalation patterns could get a bigger hit: “Inhale for like 10–15 s, you’re going to get a huge cloud. More nicotine. And some people might be using that [ENDS device] specifically for those purposes”.

Some described holding nicotine levels constant when mixing e-liquid. “I don’t really deviate from the [nicotine] strength very much, especially when it comes to vape pens. […] What I always change up though over periods of time is the flavor” (male, 22). Thus, some users preferred consistent nicotine levels as they changed flavors, indicating more interest in experimenting with flavor than nicotine concentration.

#### 3.1.2. Adding CBD and THC: “Half CBD, Half Nicotine”

Participants repeatedly mentioned using cannabis with ENDS, usually for perceived health benefits. Modifications included making cannabis tinctures for vaping, mixing cannabis with nicotine e-liquids, and using retail cannabis products with ENDS devices. One participant recounted how her family made tinctures from flower cannabis: “They have their herbal infusers, that they’re making these tinctures from, and they’re going ahead and adding it to their vapor and they’re vaping away” (female, 44). When asked why her family did this, she replied: “THC you know... It works for some people in terms of their pain and being able to sleep, and those things, so if those health benefits outweigh whatever char or whatever could be going to their lungs”.

Participants talked about mixing cannabis products with nicotine e-liquids. Some occasionally added small amounts of CBD. “You add a little bit to your tank or you can drip a little bit and it’ll help relax you a little bit, and then you can go back to vaping your regular way” (male, 29). Others regularly mixed CBD with nicotine. “I usually fill my tank mostly with just regular e-liquid, e-juice, nicotine. And then just put a little bit of CBD in it and kind of mix it together” (male, 26). Overall, mixing cannabis products with nicotine e-liquids was common, although mixture ratios varied among users.

Some participants preferred to keep cannabis and nicotine separate using a practice called “carting”, where they switched pre-filled nicotine or cannabis cartridges (i.e., “carts”) on ENDS devices. “People that buy like the pre-filled CBD cartridges just because they’re already pre-filled and you’re not mixing them with the nicotine”, said one participant (female, 30). This participant explained that keeping nicotine and CBD separate was necessary so the CBD was not tainted by “additives” in nicotine e-liquids. One participant described how retail cannabis cartridges were manufactured for carting with ENDS devices. “So, they [the vape shop] have the marijuana carts that also fit onto that [ENDS device], which is why it’s much easier to use the [device]” (male, 28). Cannabis products were described by participants as widely available at vape shops and easily added to ENDS devices, with CBD products routinely marketed as therapeutics with health benefits.

#### 3.1.3. Adjusting Wattage/Voltage: “I Really Enjoy Cranking Up the Ohms”

Participants used controls designed to adjust wattage/voltage to generate more vapor, perform vape tricks, and increase throat hits. “It’s also easier when you have a refillable one [ENDS], that you can increase the voltage, so you get a bigger cloud for your tricks” (female, 21). Another participant described modifying wattage/voltage to perform vape tricks called “donuts”, “little tornadoes”, and “dust waves”. One participant modified the wattage/voltage for a stronger hit. “I like to make it [the ohms or the voltage] really, really high and get a really powerful, deep hit” (male, 26). Participants regularly used device controls to modify wattage/voltage on their ENDS devices.

### 3.2. Motivations to Modify: Device Maintenance

Participants modified ENDS as part of routine maintenance so ENDS functioned well, provided satisfactory user experiences, and lasted longer. The most discussed maintenance modifications were maintaining coils and changing or re-charging batteries.

#### 3.2.1. Maintaining Coils: “You Have to Change the Coils out Every Once in a While”

Participants discussed how coil changing was considered normal, regular maintenance, necessary to use and enjoy ENDS devices. Specifically, participants described changing out old coils for newer ones (e.g., store-bought coils or coils wrapped by hand) or cleaning dirty coils to extend the coil’s life and prevent bad taste. Unpleasant taste was mentioned often and referred to as puffing on, sipping on, or sucking on “burnt”. One participant explained: “If you’re too lazy to replace the coil in time, it will almost feel like you’re doing burnt” (male, 36). Discussions revealed that coil replacements were necessary and that inexperienced users would eventually have to learn to modify coils if they used devices that required coil maintenance.

#### 3.2.2. Changing Batteries: “The Only Modification Is Just Getting a Spare Battery”

Modifying the battery for maintenance was limited due to the known risks of battery malfunctions. The most common modification activities could be carried out safely, such as charging one battery when using another. Participants stressed the importance of battery maintenance: “The battery is the foundation of the whole product, because if it keeps overheating and if the device doesn’t stay charged, then the coil and the juices are useless to you” (male, 36). Although participants recognized the importance of modifying for battery maintenance, they often stressed the risks of tinkering with batteries and other parts of ENDS devices, and listed many reasons not to do so.

### 3.3. Motivations to Modify: Simplicity of Design

#### Improved Modifiability: “It’s Not as Complicated as It Once Was”

Product characteristics of earlier ENDS devices were more difficult to modify, making some modifications—such as changing coils—easier in newer devices. Participants compared past devices to newer alternatives. “Three to four years ago, you had to get actual cotton and put it through this little coil-looking thing, and it was kind of hard. So now they came out with a new one [manufactured coil] that you just twist on” (female, 21). Another participant recounted, “Yeah, initially there was no customizing. You would just buy the flavor. There would be no adjustments, no filters” (male, 29). Participants described early ENDS devices as “awful” and described how routine modification maintenance (e.g., changing the coil) was much easier now than in the past. Newer devices with “self-contained” batteries also eliminated the need to “mess with” multiple batteries.

Easier-to-customize ENDS devices was cited for the decline of some modification practices. Participants used the term *modding* to describe intricate modifications “above and beyond what the manufacturer recommends” (male, 28). Examples of modding included making their own coil or altering a device not designed for dripping to be used for dripping. According to participants, the availability of more customizable devices was contributing to a decline in modding. One participant described this shift among peers: “It’s definitely been years, or at least a year, I would say, since the last person who I know that was really into it stopped” (female, 21). Thus, newer ENDS devices have made modifications such as coil replacement easier and more common while making more intricate modding, such as building a coil, less common.

### 3.4. Motivations Not to Modify: Perceived Risk

Participants recognized that ENDS modifications were potentially dangerous, and perceived risks were cited as motivation *not* to perform specific modifications, such as altering the battery. Adverse physical reactions were also listed as reasons not to modify.

#### 3.4.1. Battery Explosions

The most mentioned reason not to modify ENDS was the potential for battery explosion. Participants detailed the technical difficulty of battery modification, “Getting into battery, is at the very least entry-level electrical repair” (male, 28). Others described hearing about explosions. “You hear all the horror stories of people’s big batteries exploding and blowing up people’s faces” (female, 30). One participant’s friend was injured from a battery explosion. “He had to have plastic surgery just to get by because it exploded in his pocket. It burned him so bad he had to have skin grafts” (male, 28). Fearful talk about battery explosions was common and repeatedly cited as a reason not to modify ENDS’ batteries.

#### 3.4.2. Adverse Physical Reactions

It was common for those who mixed e-liquids to describe mixes that tasted bad or caused nausea. One participant described physical illness. “I did that [mixed] with vanilla and this mango flavor, and I got very sick from it” (male, 28). Interestingly, later in the discussion, this participant said he would mix e-liquids again: “I’ll do it because due to the fact that I’ve seen now, there’s numerous more flavors and now there’s more videos and people, whose experiences, know how to do it”. This exchange provided one example of how users weigh benefits against risks when modifying ENDS and how modification behaviors change over time based on new information and product availability.

### 3.5. Sources of Information for Modifications

#### 3.5.1. In-Person and Social Media

Participants cited other users and social media for information on ENDS modifications. Vape shop employees demonstrated “how to put the coils in if we’re brand new to vaping. They show us how to fill the tanks, how to prime the coil and how the mod actually works” (female, 24). Another participant relied on friends unless the modification was too technical, in which case he turned to social media. “There has been situations, especially when I was first getting into replacing the coils I used to depend on YouTube videos” (male, 22).

YouTube was repeatedly mentioned for information about ENDS modifications. “Doing research on YouTube and things like that, I can find recommendations on flavors made by numerous people that can recommend me to do this and be safe”, said one participant (male, 28). Another explained learning about modifying: “Making sure the coils and everything stay good, that was difficult. But I watched YouTube videos on how to do it” (female, 30). Participants also mentioned Facebook and Reddit as sources of modification information. Thus, social interactions—either face-to-face or online—were common sources of information about modifying ENDS devices.

#### 3.5.2. Manufacturer Instructions

Participants felt ENDS instructions did not provide adequate information to repair or modify devices, and they rarely read the instructions. “An [instruction] manual isn’t going to say, ‘Hey, sometimes your coil may be bent, but first of all you have to use tweezers and re-bend it’” (female, 30). One participant tried going to a manufacturer’s website to find ENDS information, only to turn to YouTube in frustration:

“I went onto [manufacturer’s] site directly. […] I had a little chap say, ‘How may I help you?’ And I said, ‘I want to know more about the device.’ And they were, ‘Right, you can purchase it at Smoke Shops, vape shops online.’ And I said, ‘How to use it?’ And then I went back to the YouTube, I felt embarrassed” (female, 52).

## 4. Discussion

This study describes the ENDS modification behaviors of regular ENDS users to understand what motivates them to modify devices and how perceived risks of modifications influence behaviors. Our research adds to the literature [12,13,14] by showing that regular ENDS users regularly perform modifications on their devices in ways intended and unintended by manufacturers. These findings differ from the previous research of ENDS enthusiasts who indicated that ENDS modifications were becoming less common with consumers. Findings on specific modifications—such as coil replacement—align with common modifications in prior research [13]. Modifying to use cannabis indicates future areas of research. Overall, results highlight the ongoing need to monitor trends in modification behaviors, particularly given the severe health risks of some modifications [15,16,17,18,19,20], and ongoing regulatory uncertainty about ENDS in the United States.

Modifications were common among regular users in our study, and not a passing fad, as we had found with interviews with enthusiasts [12]. Furthermore, some of the modifications mentioned (e.g., changing coils, mixing flavors, changing batteries, and altering wattage) could be classified as intended by manufacturers, whereas other modifications (e.g., cannabis; discussed below) could not. Both intended and unintended modifications can be harmful. For example, higher wattage (an intended modification) yields higher levels of harmful aldehydes in aerosols [17,18,19,20]; wiring more powerful batteries (an unintended modification) can lead to device explosions [15,16]. Therefore, regulatory agencies need a better understanding of consumer behaviors to take action to address the risk resulting from modification behaviors. Our data suggest that intended and unintended modifications are common, and should continually be monitored, especially considering potential dangers from modifying. When discussing modification dangers, participants primarily focused only on perceived risk of battery alterations, which can lead to explosions. Although a few mentioned feeling sick from homemade e-liquid mixes, participants did not elaborate on other harms from modifications. The lack of knowledge of health harms of modifications might be precipitated by the mostly positive portrayal of modifications on social media, where many ENDS users find their information [13]. ENDS users should be educated about possible health risks of modifications, particularly in the evolving ENDS market.

We found that ENDS modifications were changing alongside market availability, with newer devices making some modifications (e.g., coil replacement) easier and making more intricate modifications (e.g., building coil from scratch) less common. According to the participants, newer devices eliminated the need for some modifications viewed as dangerous (such as wiring new batteries). However, newer devices might pose risks to users, as can happen when device controls are used to over-heat coils and cause higher levels of toxic emissions in aerosol clouds [17,18]. The continuously changing design and technology of ENDS devices make detecting and reporting any adverse effects related to ENDS modification a fundamental public health issue. Consequently, it is crucial to warn consumers about the potential health harms related to ENDS modifications. Therefore, efforts to continue monitoring the modifications and educating users on the potential harms of modifications are needed.

A critical area of future research is the use of cannabis with ENDS devices. The practice of carting was mentioned frequently in focus groups and touches on several regulatory and legal questions about the commercial production of cannabis products for ENDS devices, especially given the increasing legalization of retail cannabis. Devices advertised for use with substances that are not made or derived from tobacco, such as cannabis, but are readily used with tobacco, may be subject to tobacco regulation if the product design is conducive to dual-use or commonly used with both substances. Although the legal issues remain to be determined, understanding the interchangeability of substances and whether the product can be modified to allow dual use is critical in determining whether it is subject to tobacco regulatory authority. This study adds to the literature by documenting the everyday use of cannabis with ENDS devices. Tobacco control and cannabis are likely to intersect more and more as cannabis becomes legal in additional U.S. jurisdictions and sold for use with ENDS devices. The intersection of cannabis and ENDS is a critical public health domain that deserves urgent attention, especially given the links between vaping cannabis and severe lung injury [20,25].

### Limitations

Due to the qualitative design, the generalizability of our results is limited. The majority of focus groups were during the onset and continued COVID-19 pandemic in the United States, and most groups used videoconferencing technology, which may have affected the results. Conducting focus groups over remote technology could have affected conversational norms in face-to-face discussions, such as maintaining eye contact or not talking over other participants. Although remote technology offers a call-in feature for those without reliable internet access, the use of videoconferencing software could have led to selection bias based on access to reliable internet.

## 5. Conclusions

Current ENDS users modified devices to improve user experience. Users were aware of the dangers of modifying, and risk perceptions guided modification behaviors. They also weighed the benefits of modifying against the perceived risk, and these considerations led to changes in modification behavior over time as the product landscape and information about the dangers of modifying changed. These results indicate that modifications of ENDS are ongoing and evolving among users. Due to the possibility of additional health risks due to products that can be consumer-modified, the FDA should evaluate whether companies perform adequate evaluations of human factors, including normal use and foreseeable misuse conditions, when determining whether new products receive market authorization. However, FDA regulatory actions to limit the variability of ENDS available on the market should also consider how this may encourage more user modification that could lead to unanticipated consequences.

## Figures and Tables

**Table 1:** Characteristics of focus group participants (*N* = 32).

Demographic Characteristics	*n* (%)
Sex	
Male	17 (53.1)
Female	15 (46.9)
Age	
18–29	18 (56.3)
30–44	13 (40.6)
45–59	1 (3.1)
Race	
Asian	3 (9.4)
Black or African American	8 (25.0)
White	21 (65.6)
Spanish, Hispanic, or Latinx	4 (12.5)
**Tobacco use**	
Never smoker, current ENDS user ^a^	7 (21.9)
Former smoker, current ENDS user ^b^	7 (21.9)
Current smoker, current ENDS user ^c^	18 (56.3)
**Vaping frequency** ^d^	
Every day	23 (71.9)
Some days	9 (28.1)

^a^ Among never smokers, 6 reported having never smoked tobacco cigarettes and 1 reported having ever smoked tobacco cigarettes but not having smoked 100 cigarettes in their lifetime. ^b^ Former smokers had smoked over 100 cigarettes in their lifetime, but not currently smoking (Selecting “not at all” in response to “*Do you now smoke cigarettes every day, some days, or not at all?*”). ^c^ Current smokers had smoked over 100 cigarettes in their lifetime and were currently smoking “every day” or some days”. ^d^ Measured as “Do you now use electronic vapor products every day, some days, rarely, or not at all?” (No participant selected “rarely” or “not at all”).

## Data Availability

The data underlying this article will be shared on reasonable request to the corresponding author.

## References

[1] National Academies of Sciences Engineering Medicine (2018). Public Health Consequences of E-Cigarettes.

[2] U.S. Department of Health and Human Services (2016). E-Cigarette Use Among Youth and Young Adults: A Report of the Surgeon General.

[3] McMillen R.C., Gottlieb M.A., Shaefer R.M.W., Winickoff J.P., Klein J.D. (2014). Trends in electronic cigarette use among US adults: Use is increasing in both smokers and nonsmokers. Nicotine Tob. Res..

[4] Cobb N.K., Abrams D.B. (2014). The FDA, e-cigarettes, and the demise of combusted tobacco. N. Engl. J. Med..

[5] Henningfield J.E., Zaatari G.S. (2010). Electronic nicotine delivery systems: Emerging science foundation for policy. Tob. Control.

[6] Etter J.-F., Bullen C., Flouris A.D., Laugesen M., Eissenberg T. (2011). Electronic nicotine delivery systems: A research agenda. Tob. Control.

[7] Blundell M., Dargan P., Wood D. (2017). A cloud on the horizon–a survey into the use of electronic vaping devices for recreational drug and new psychoactive substance (NPS) administration. QJM Int. J. Med..

[8] Glasser A.M., Collins L., Pearson J.L., Abudayyeh H., Niaura R.S., Abrams D.B., Villanti A.C. (2017). Overview of electronic nicotine delivery systems: A systematic review. Am. J. Prev. Med..

[9] Zhu S.-H., Sun J.Y., Bonnevie E., Cummins S.E., Gamst A., Yin L., Lee M. (2014). Four hundred and sixty brands of e-cigarettes and counting: Implications for product regulation. Tob. Control.

[10] Berg C.J. (2018). Vape shop location and marketing in the context of the Food and Drug Administration regulation. Public Health.

[11] Brown C.J., Cheng J.M. (2014). Electronic cigarettes: Product characterisation and design considerations. Tob. Control.

[12] Li Y., Fairman R., Churchill V., Ashley D., Popova L. (2020). Users’ modifications to electronic nicotine delivery systems (ENDS): Interviews with ENDS enthusiasts. Int. J. Environ. Res. Public Health.

[13] Massey Z., Li Y., Holli J., Churchill V., Yang B., Henderson K., Ashley D., Huang J., Popova L. (2020). Modifications to electronic nicotine delivery systems: Content analysis of YouTube videos. J. Med. Internet Res..

[14] Alexander J.P., Williams P., Coleman B., Johnson S.E. (2018). A qualitative examination of the ENDS experience by device type: Cigalike and tank users’ attitudes, beliefs, and behaviors. Tob. Regul. Sci..

[15] Rossheim M.E., Livingston M.D., Soule E.K., Zeraye H.A., Thombs D.L. (2019). Electronic cigarette explosion and burn injuries, US Emergency Departments 2015–2017. Tob. Control.

[16] Rossheim M.E., Soule E.K., Thombs D.L., Barnett T.E., Livingston M.D., Gimm G., Emechebe O.C. (2020). Electronic cigarette explosion and burn injuries. Tob. Regul. Sci..

[17] Kosmider L., Sobczak A., Fik M., Knysak J., Zaciera M., Kurek J., Goniewicz M.L. (2014). Carbonyl compounds in electronic cigarette vapors: Effects of nicotine solvent and battery output voltage. Nicotine Tob. Res..

[18] Talih S., Balhas Z., Eissenberg T., Salman R., Karaoghlanian N., El Hellani A., Baalbaki R., Saliba N., Shihadeh A. (2014). Effects of user puff topography, device voltage, and liquid nicotine concentration on electronic cigarette nicotine yield: Measurements and model predictions. Nicotine Tob. Res..

[19] Centers for Disease Control and Prevention Outbreak of Lung Injury Associated with E-Cigarette Use, or Vaping. https://www.cdc.gov/tobacco/basic_information/e-cigarettes/severe-lung-disease.html.

[20] King B.A., Jones C.M., Baldwin G.T., Briss P.A. (2020). The EVALI and youth vaping epidemics—Implications for Public Health. N. Engl. J. Med..

[21] United States Department of Health and Human Services NIoH, National Institute on Drug Abuse, United States Department of Health and Human Services, Food and Drug Administration, Center for Tobacco Products (2021). Population Assessment of Tobacco and Health (PATH) Study [United States] Restricted-Use Files.

[22] Kong G., Morean M.E., Bold K.W., Wu R., Bhatti H., Simon P., Krishnan-Sarin S. (2020). Dripping and vape tricks: Alternative e-cigarette use behaviors among adolescents. Addict. Behav..

[23] Krishnan-Sarin S., Morean M., Kong G., Bold K.W., Camenga D.R., Cavallo D.A., Simon P., Wu R. (2017). E-cigarettes and “dripping” among high-school youth. Pediatrics.

[24] Harrell P.T., Eissenberg T. (2018). Automated dripping devices for vapers: RDTAs, bottomfeeders, squonk mods and dripboxes. Tob. Control.

[25] Choi H., Lin Y., Race E., Macmurdo M.G. (2021). Electronic cigarettes and alternative methods of vaping. Ann. Am. Thorac. Soc..

[26] Breitbarth A.K., Morgan J., Jones A.L. (2018). E-cigarettes—An unintended illicit drug delivery system. Drug Alcohol Depend..

[27] Lim C.C.W., Leung J., Chung J.Y.C., Sun T., Gartner C., Connor J., Hall W., Chiu V., Tisdale C., Stjepanović D. (2021). Content analysis of cannabis vaping videos on YouTube. Addiction.

[28] Neergaard M.A., Olesen F., Andersen R.S., Sondergaard J. (2009). Qualitative description–the poor cousin of health research?. BMC Med. Res. Methodol..

[29] Sandelowski M. (2000). Whatever happened to qualitative description?. Res. Nurs. Health.

[30] Coenen M., Stamm T.A., Stucki G., Cieza A. (2012). Individual interviews and focus groups in patients with rheumatoid arthritis: A comparison of two qualitative methods. Qual. Life Res..

[31] O’Brien E.K., Nguyen A.B., Persoskie A., Hoffman A.C. (2017). US adults’ addiction and harm beliefs about nicotine and low nicotine cigarettes. Prev. Med..

[32] Palinkas L.A., Horwitz S.M., Green C.A., Wisdom J.P., Duan N., Hoagwood K. (2015). Purposeful Sampling for Qualitative Data Collection and Analysis in Mixed Method Implementation Research. Adm. Policy Ment. Health.

[33] QSR International Pty Ltd. (2020). NVivo (Released March 2020). https://www.qsrinternational.com/nvivo-qualitative-data-analysis-software/home.

